# Compensatory lung growth after bilobectomy in emphysematous rats

**DOI:** 10.1371/journal.pone.0181819

**Published:** 2017-07-27

**Authors:** Francine Maria Almeida, Beatriz Mangueira Saraiva-Romanholo, Rodolfo Paula Vieira, Henrique Takachi Moriya, Ana Paula Ligeiro-de-Oliveira, Fernanda DTQS Lopes, Hugo C. Castro-Faria-Neto, Thais Mauad, Milton Arruda Martins, Rogerio Pazetti

**Affiliations:** 1 Faculdade de Medicina, Universidade São Paulo, LIM20, São Paulo, Brazil; 2 IAMSPE–UNICID, São Paulo, Brazil; 3 Brazilian Institute of Teaching and Research in Pulmonary and Exercise Immunology (IBEPIPE), School of Medical Sciences of Sao Jose dos Campos Humanitas and Universidade Brazil, São Paulo, Brazil; 4 Biomedical Engineering Laboratory (LEB), USP, São Paulo, Brazil; 5 Institute Oswaldo Cruz (Laboratory of Immunopharmacology), Rio de Janeiro, Brazil; 6 Pathology (LIM05), FMUSP, São Paulo, Brazil; 7 Hospital das Clinicas HCFMUSP, Faculdade de Medicina, Universidade Sao Paulo, Cardiopneumology (LIM61), São Paulo, Brazil; University of Giessen Lung Center, GERMANY

## Abstract

Lung volume reduction surgery (LVRS) is an option for emphysematous patients who are awaiting lung transplantation. LVRS reduces nonfunctional portions of lung tissues and favors the compensatory lung growth (CLG) of the remaining lobes. This phenomenon diminishes dyspnea and improves both the respiratory mechanics and quality of life for the patients. An animal model of elastase-induced pulmonary emphysema was used to investigate the structural and functional lung response after LVRS. Bilobectomy was performed six weeks after elastase instillation. Two weeks after bilobectomy, CLG effects were evaluated by lung mechanics and histomorphometric analysis. After bilobectomy, the emphysematous animals presented decreased mean linear intercepts, increased elastic fiber proportion, and increased alveolar surface density, total volumes of airspace, tissue and respiratory region and absolute surface area. We conclude that bilobectomy promoted CLG in emphysematous animals, resulting in alveolar architecture repair.

## Introduction

Pulmonary emphysema is characterized by permanent destruction of the alveolar walls leading to (i) airspace enlargement, (ii) loss of elastic recoil, (iii) decreased gas-exchange surface area, (iv) pulmonary hyper distension, and (v) increased respiratory effort [1]. Lung volume reduction surgery (LVRS) is an alternative surgical approach for emphysematous patients awaiting lung transplantation. LVRS can result in immediate improvement of quality of life and exercise capacity, as well as reduction in the mortality rate [2]. Besides the expansion of normal collapsed alveolar units, compensatory lung growth (CLG) is one of the most acceptable hypotheses for this life quality improvement. During the adaptive event, alveolar epithelial cell proliferation in the remaining lobes is associated with increased lung weight [3] and network reconstitution [4].

However, it is still unclear whether and how LVRS increases or induces CLG, even in emphysematous lung tissue [5]. Studies measuring morphometric characteristics such as alveolar surface density and total volume of respiratory tissue can help to shed light on the histological changes in emphysematous lungs after LVRS.

## Methods

This study was approved by our institutional research committee (CAPPesq 0750/09). Animals were fed and humanely taken care of according to the National Institute for Health Guide for the Care and Use of Laboratory Animals [6].

### Experimental groups

The Central Animal Facility of the University of Sao Paulo provided 32 male Wistar rats that were 6–8 weeks old, which were maintained at standard conditions. Animals were randomized into four groups (n = 8 each) according to (i) treatment with saline (Sal) or elastase (Ela) and (ii) surgical procedure sham or lobectomy/LBX: Sal+Sham; Sal+LBX; Ela+Sham; Ela+LBX (S1A Fig).

### Emphysema induction

Animals were anesthetized (isoflurane 5%), orotracheally intubated (catheter 14G), and mechanically ventilated (flexiVent® SCIREQ, Montreal, Canada) at 120 breaths per minute with a tidal volume of 10 mL/kg and PEEP of 2 cmH_2_O. Porcine pancreatic elastase was instilled intratracheally for around two minutes (5 UI/100 g diluted in 600 μL of saline solution). The saline groups received only vehicle (S1B Fig).

### Surgery

Six weeks after instillation, the animals were anesthetized, intubated, and mechanically ventilated as described in section 2.2. General anesthesia was maintained with 2% isoflurane. After right thoracotomy, the hila of the middle and cardiac lobes were dissected and tied with a 2–0 silk ligature, and the lobes were excised. Animals received postoperative analgesia with dipyrone (100 mg/kg by gavage for 3 consecutive days). Sham groups (Sal+Sham and Ela+Sham) underwent a similar surgical procedure without the excision of any lobe. After surgery, animals were maintained in individual cages and monitored during the first six hours and then daily for two weeks.

### Respiratory mechanics

Two weeks after the surgical procedures, the animals were anesthetized, intubated, and mechanically ventilated as described in section 2.2. Quasi-sinusoidal volume perturbations (1.5 Hz with the same tidal volume and PEEP) were applied to measure the resistance and elastance of the respiratory system [7].

### Blood gases

After assessment of the respiratory mechanics, arterial blood samples (0.3 mL) were collected by left atrium puncture and analyzed using Stat Profile 10 (Nova Biomedical, EUA). Immediately after blood collection, the animals were euthanized under anesthesia by total section of the abdominal aorta, and the heart-lung bloc was excised from the thoracic cavity.

### Right ventricle hypertrophy

Right ventricle (RV) hypertrophy was assessed as an index of pulmonary hypertension. The RV free wall was excised from the heart and weighed, as was the left ventricle wall (LV) + interventricular septum (S). The ratio of the RV weight to body weight (BW) and that of RV to LV + S were used as indexes of RV hypertrophy [8].

### Bronchoalveolar lavage fluid (BALF)

BALF was selectively performed on the left lung by instillation of 5 mL of saline solution. After centrifugation (800 g/10 min/5°C) and removal of the supernatant, the cell pellet was resuspended with 5 mL of PBS, and the total number of cells per mL was counted in a Neubauer hemocytometer chamber. In addition, 300 cells were counted in slides prepared using a cytocentrifuge (Cytospin-2, Shandon Instruments Sewickley, USA) and stained with Diff Quick (NEWPROV).

### Lung weight and volume

The right lower lobe was dried and weighed and then intratracheally instilled with 4% paraformaldehyde under constant pressure (25 cmH_2_O). The trachea was then tied, and lung volume was obtained by immersion in a measuring cylinder containing paraformaldehyde [9].

### Histomorphometry

After lung volume measurement, the right lower lobe was kept in paraformaldehyde solution for 24 h. Next, it was processed for histological analysis (paraffin bloc containing 3 sections of 5 μm thickness per animal). The sections were stained with hematoxylin-eosin, resorcin-fuchsin, and picrosirius.

#### Hematoxylin-eosin

The mean linear intercept (Lm) was determined by the interception number count between the alveolar septa and reticule straight (Weibel reticule of 50 straight and 100 points) with 15 random fields per slides [10]. Mononuclear (MN) and polymorphonuclear (PMN) cell density were evaluated on alveolar septa by conventional morphometric techniques [11, 12]. A three-level sampling technique (S1 Table) was used as previously described by Fernandez et al. [9, 13]. The alveolar surface density (Sv), total volume of the respiratory region (TVvr), total volume of the respiratory airspace (TVra), total volume of the respiratory tissue (TVrt) and absolute surface area were measured.

#### Resorcin-fuchsin and picrosirius

Elastic and collagen fibers in the lung parenchyma were stained by resorcin-fuchsin and picrosirius, respectively, and the percentages of fibers per tissue area were quantified by image analysis (Image-Pro Plus, 4.5.0.29 for Windows, Media Cybernetics, Silver Spring, MD) using 15 images per slide. Images were acquired with a light microscope (400x magnification) and a digital camera connected to a computer (BM4000B, Leica, Germany) [12]. Histomorphometric analysis was performed by two blinded researchers.

### Vascular endothelial growth factor (VEGF)

The right upper lobes were homogenized in protein extraction solution. Protein quantification was performed by the BCA method (BCA Protein Assay kit). Samples containing 50 μg of protein were denatured, run on SDS-PAGE gel electrophoresis, and transferred to a nitrocellulose membrane. Protein expression was subsequently detected by incubation with rabbit polyclonal primary antibodies against VEGF (1:500; sc-507; Santa Cruz Biotechnology) at 4 C for 12 hours. Binding of the primary antibodies was detected with secondary antibodies conjugated to horseradish peroxidase, and the protein bands were detected by ImageJ software (National Institutes of Health) [14]. A work-flow scheme in the supporting information (S2 Fig) indicates the analysis performed on each pulmonary lobe.

### Statistical analysis

Data are presented as mean ± standard deviation (SD). The normal distribution in each group and homogeneity of variances between groups were evaluated with the Shapiro-Wilk and Levene tests, respectively. We used two-factor analysis of variance for intergroup comparison of all parameters evaluated at the time of euthanasia. In the event of statistical difference (p < 0.05), we used the Bonferroni post hoc test for multiple comparisons of means.

## Results

### Lm

**Fig 1:**
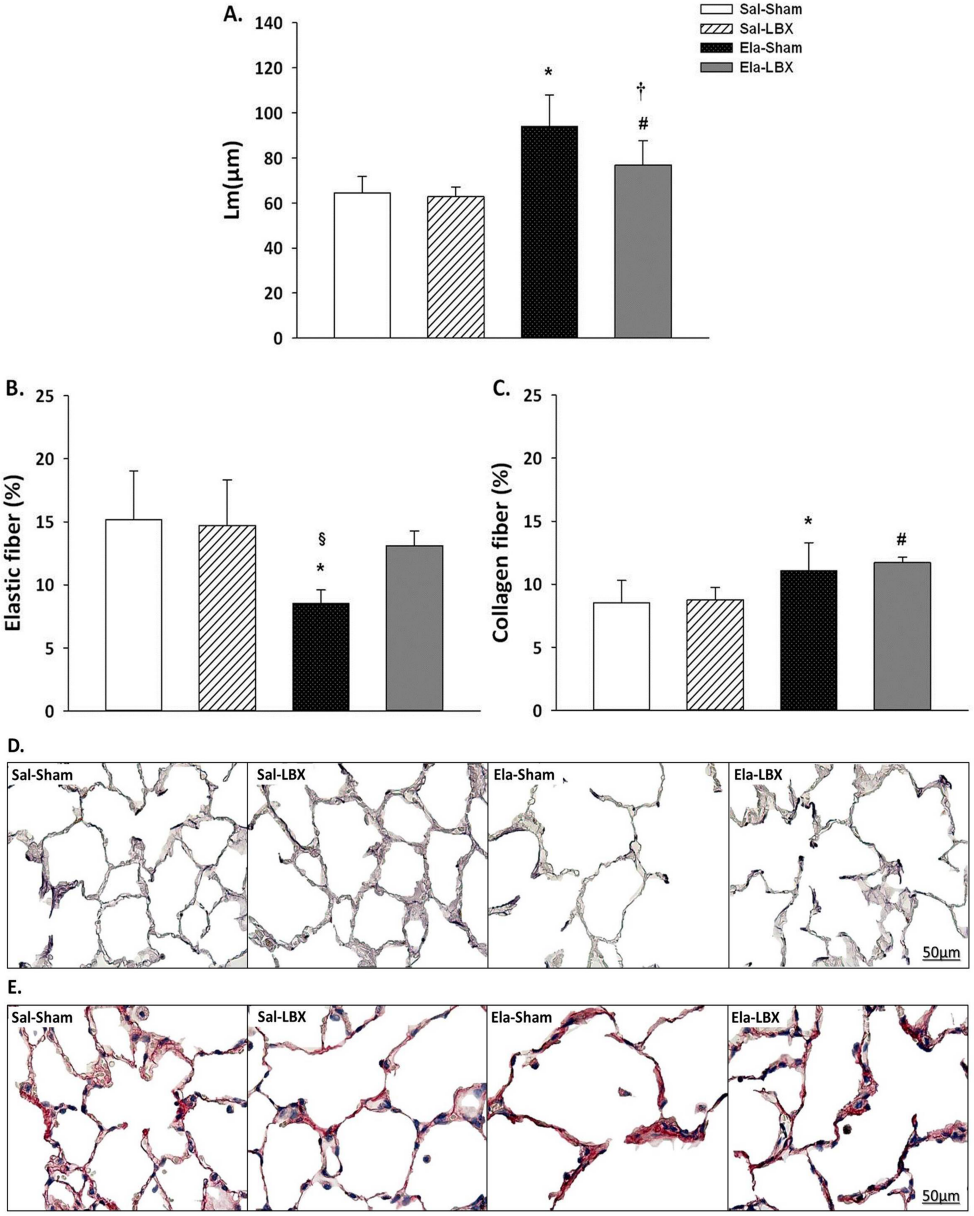
Morphometric parameters and images analysis in lung parenchyma. (*A)* Mean linear intercept (Lm_,_ μm); (*B)* Elastic fibers per tissue area (%); (*C)* Collagen fibers per tissue area (%); (D) Photomicrographs of the lung parenchyma stained by resorsin-fuchsin (400x); (E) Photomicrographs of the lung parenchyma stained by picrosirius (400x). Values are expressed as mean ± SD. (*) vs. Sal+Sham; (#) vs. Sal+LBX; (†) vs. Ela+Sham; (§) vs. Ela+LBX; p < 0.05.

Lm was larger in Ela+Sham (94.1 ± 13.8 μm) and Ela+LBX (76.8 ± 10.9 μm) in comparison to Sal+Sham (64.5 ± 7.2 μm) and Sal+LBX (62.7 ± 4.3 μm), respectively (p ≤ 0.010). The Ela+LBX group presented smaller Lm than the Ela+Sham group (p = 0.002) (Fig 1A).

### Elastic and collagen fibers

The percentage of elastic fibers per tissue area was lower in Ela+Sham (8.5 ± 1.1%) than in Sal+Sham (15.2 ± 3.8%) and Ela+LBX (13.1 ± 1.2%) (p < 0.001) (Fig 1B and 1D). The percentage of collagen fibers was higher in Ela+Sham (11.1 ± 2.2%) and Ela+LBX (11.7 ± 0.4%) than in Sal+Sham (8.5 ± 1.7%) and Sal+LBX (8.7 ± 0.9%), respectively (p ≤ 0.015) (Fig 1C and 1E).

### Inflammation in BALF

**Fig 2:**
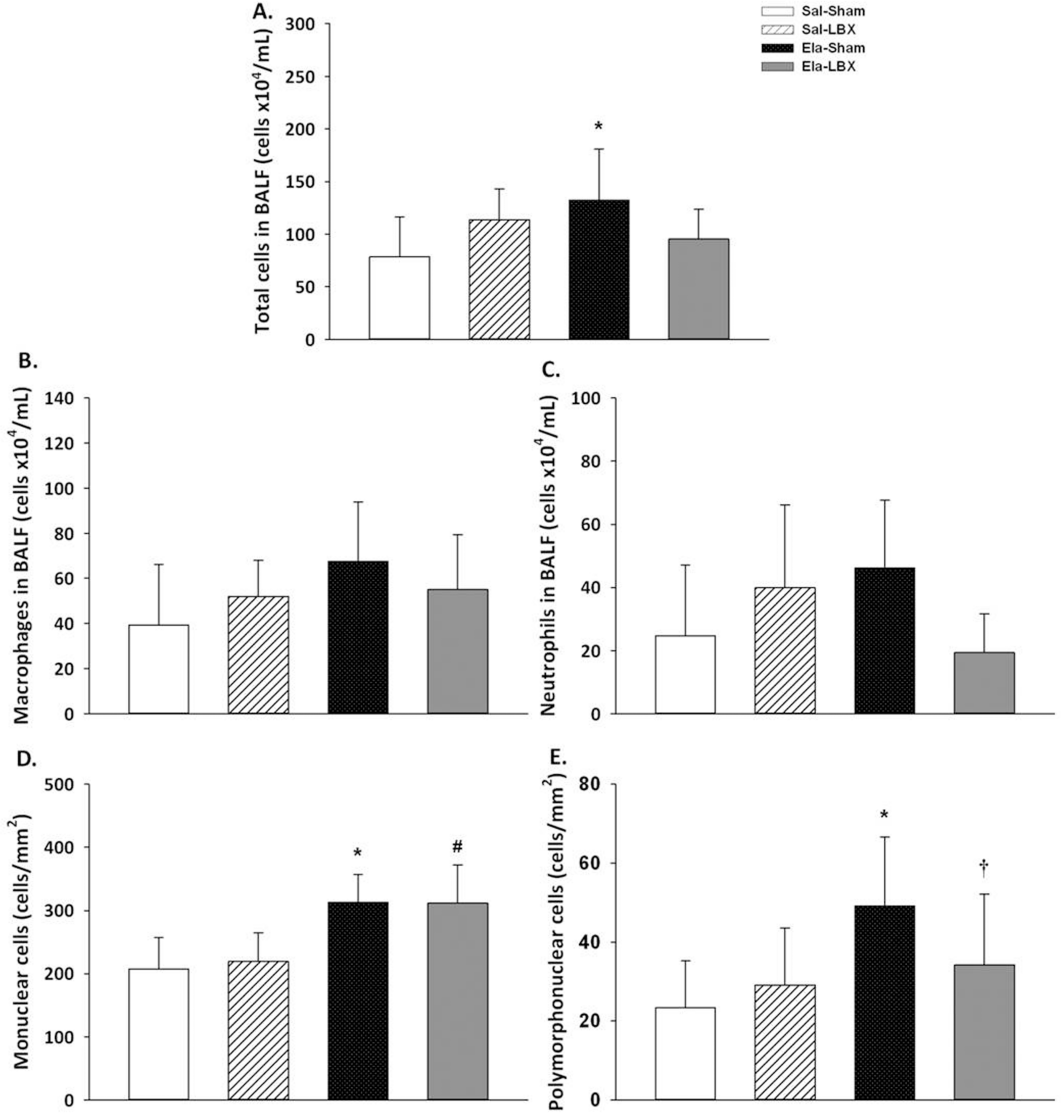
Inflammatory cells in bronchoalveolar lavage fluid and in lung tissue. (*A)* Total cells values in BALF (cells x10^4^/mL) were higher in the Ela+Sham group; (*B)* Macrophages in BALF (cells x10^4^/mL); (*C)* Neutrophils in BALF (cells x10^4^/mL); (*D)* The number of mononuclear cells in lung tissue (cells/mm^2^) was higher in elastase groups. (*E)* The number of polymorphonuclear cells in lung tissue (cells/mm^2^) was higher in the Ela+Sham group. Values are expressed as mean ± SD. (*) vs. Sal+Sham; (#) vs. Sal+LBX; (†) vs. Ela+Sham; p < 0.05.

The total number of cells was higher in Ela+Sham (132.5 ± 48.2 cells x10^4^/mL) than in Sal+Sham (78.3 ± 37.8 cells x10^4^/mL) (p = 0.045) (Fig 2A). There was no difference among groups concerning the number of macrophages and neutrophils in BALF (Fig 2B and 2C).

### Inflammation in lung tissue

Ela+Sham and Ela+LBX showed higher numbers of MN (312.5 ± 44.8 cells/mm^2^ and 311.6 ± 60.3 cells/mm^2^_,_ respectively) in comparison with Sal+Sham (207.0 ± 50.1 cells/mm^2^) and Sal+LBX (219.0 ± 45.4 cells/mm^2^) (p ≤ 0.005) (Fig 2D). The number of PMN per tissue area was higher in Ela+Sham (49.1 ± 17.4 cells/mm^2^) than in Sal+Sham (23.3 ± 11.8 cells/mm^2^) (p < 0.001), while Ela+LBX presented less PMN (34.1 ± 17.9 cells/mm^2^) in comparison with Ela+Sham (p = 0.024) (Fig 2E).

### Weight and volume of the lungs

**Fig 3:**
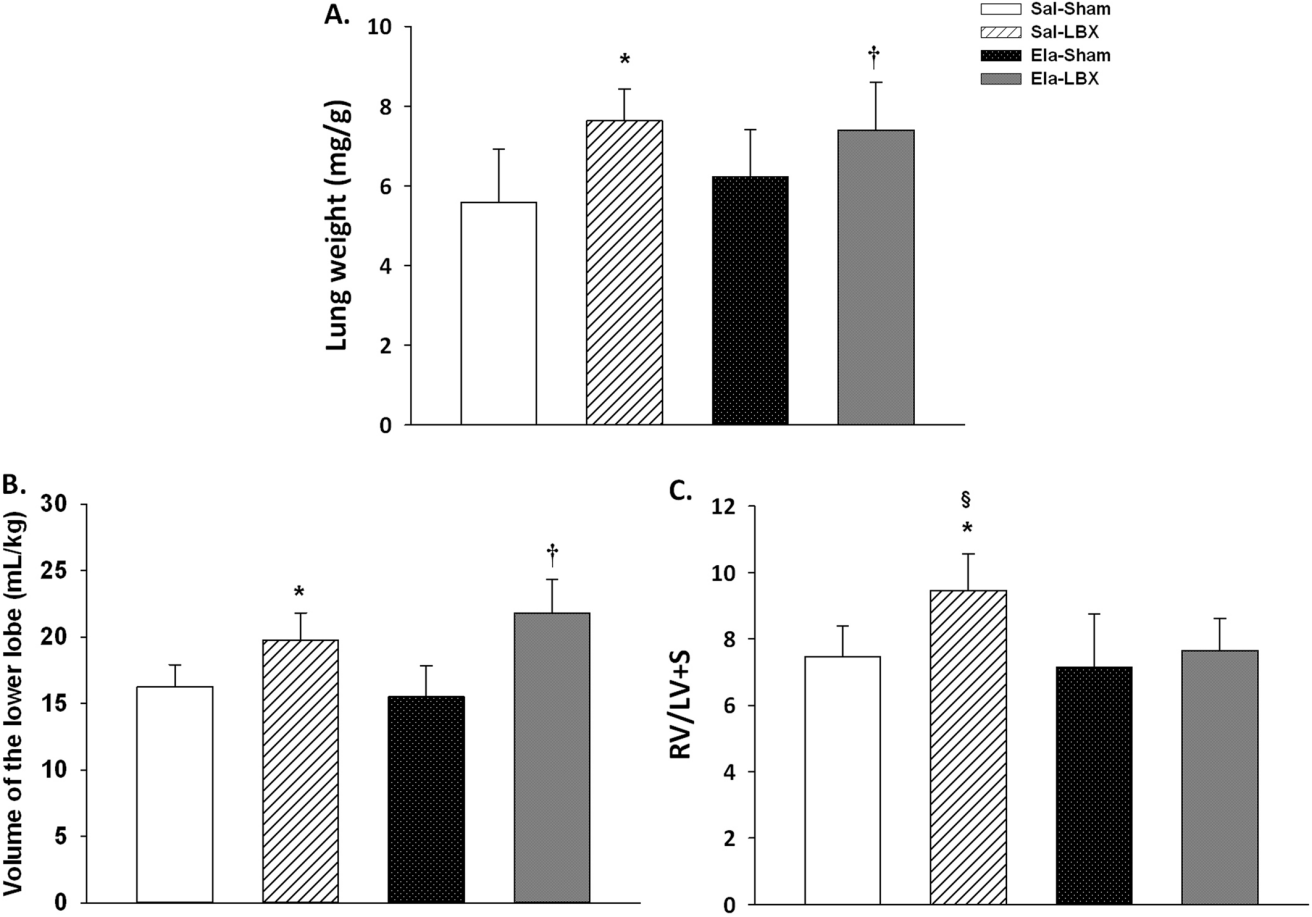
Lung weight, lung volume, and hypertension index. (*A)* Lung weight (mg/g) was higher in both Sal+LBX and Ela+LBX groups; (*B)* Volume of the lower lobe (mL/kg) was higher in Sal+LBX and Ela+LBX; (C) Right ventricle to left ventricle + septum to body weight (x10^7^ mg/Kg). This index was higher in the Sal+LBX group. Values are expressed as mean ± SD. (*) vs. Sal+Sham; † vs. Ela+Sham; (§) vs. Ela+LBX; p < 0.05.

The lung weight was higher in Sal+LBX (7.6 ± 0.8 mg/g) and Ela+LBX (7.4 ± 1.2 mg/g) in comparison to Sal+Sham (5.5 ± 1.3 mg/g) and Ela+Sham (6.2 ± 1.1 mg/g), respectively (p ≤ 0.012) (Fig 3A). The right lower lobe volume was higher in Sal+LBX (0.019 ± 0.002 mL/Kg) and Ela+LBX (0.021 ± 0.002 mL/Kg) in comparison with Sal+Sham (0.015 ± 0.002 mL/Kg) and Ela+Sham (0.015 ± 0.002 mL/Kg), respectively (p ≤ 0.002) (Fig 3B).

### Arterial blood analysis and hypertension index

The RV/LV+S ratio was higher in Sal+LBX (9.5 ± 1.1 x10^7^ mg/Kg) than in Sal+Sham (7.5 ± 0.9 x10^7^ mg/Kg) and Ela+LBX (8.4 ± 1.1 x10^7^ mg/Kg) (p ≤ 0.004) (Fig 3C). There was no difference in arterial blood analysis (S2 Table).

### Compensatory lung growth

**Fig 4:**
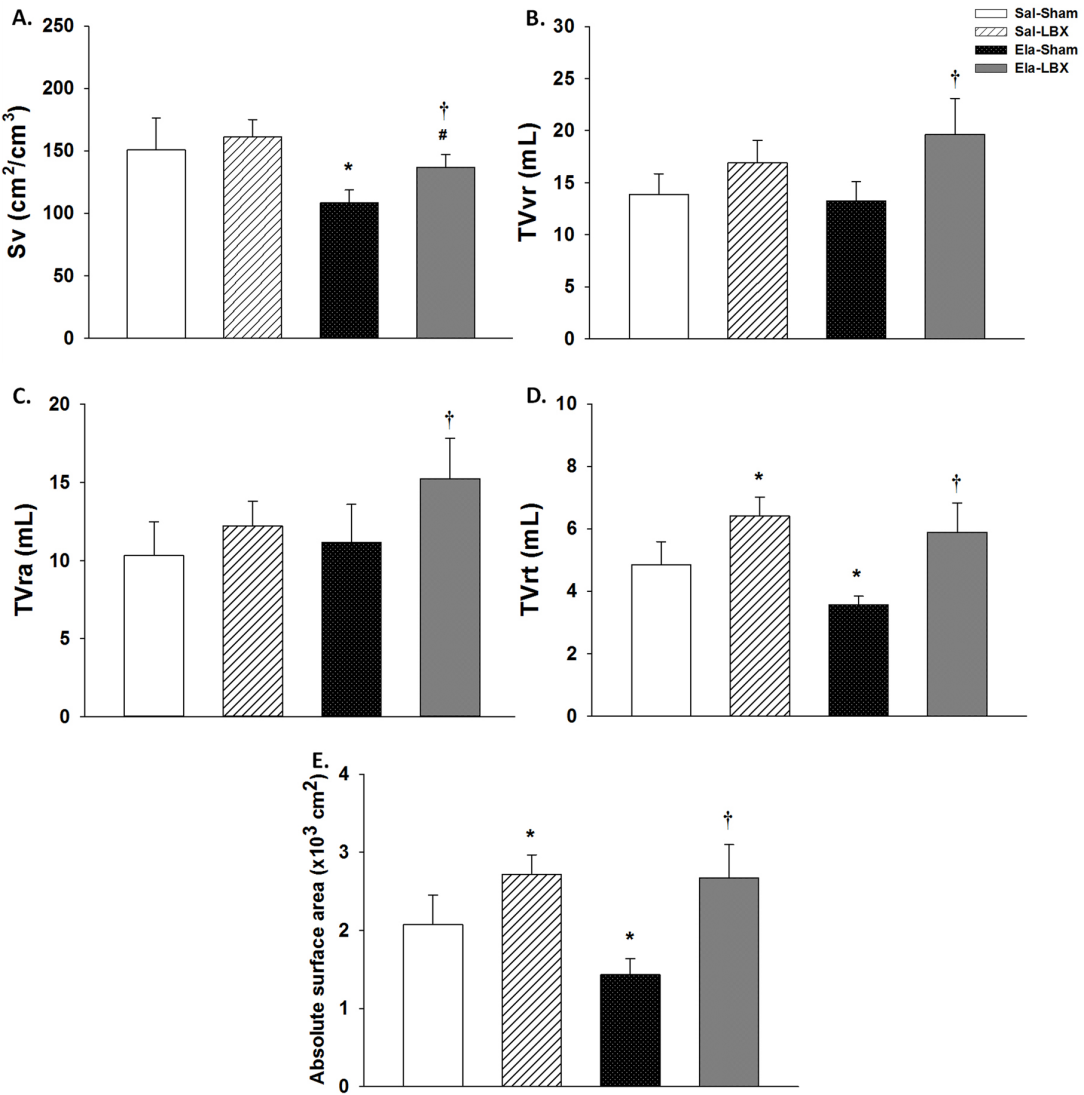
Compensatory lung growth parameters. (*A)* Alveolar surface density (cm^2^/cm^3^) was lower in elastase group; (*B)* Total volume of respiratory region (mL) was higher in the Ela+LBX group; (*C)* Total volume of respiratory airspace (mL) was higher in the elastase groups and Sal+LBX; (*D)* Total volume of respiratory tissue (mL) was higher in the Sal+LBX group, but the Ela+Sham group showed lower TVrt than the other groups; (E) Absolute surface area (x10^3^ cm^2^) was higher in LBX animals. Values are expressed as mean ± SD. (*) vs. Sal+Sham; (#) vs. Sal+LBX; (†) vs. Ela+Sham; p < 0.05.

The alveolar surface density (Sv) was higher in the operated animals in comparison with Ela+Sham group (p < 0.001) (Fig 4A).The total volume of respiratory region (TVvr) was higher in the Ela+LBX group compared with Ela+Sham (p < 0.001) (Fig 4B). There was an increase in the total volume of the respiratory airspace (TVra) of the Ela+LBX in comparison with Ela+Sham group (p ≤ 0.008) (Fig 4C). In addition, animals that underwent LBX showed higher total volume of respiratory tissue (TVrt) and absolute surface area than sham-operated animals (p < 0.001) (Fig 4D and 4E).

### VEGF levels and respiratory mechanics

There was no difference among groups concerning VEGF expression, resistance, and elastance values (S3 Fig).

## Discussion

In this study, we show that elastase-induced emphysematous animals that underwent bilobectomy presented compensatory lung growth of the remaining lobes. This growth was supported by the increase in several parameters, such as lung weight, total volume of respiratory region, alveolar surface density and absolute surface area. Then emphysematous animals had maintained their capacity of recovering gas exchange areas and neoalveolization at the same level of non emphysematous animals.

Several studies show improvements in pulmonary function, exercise capacity, and quality of life for patients underwent LVRS [2, 15–17]. However, there are limiting factors related to postoperative complications, such as tracheobronchitis and pneumonia, which are associated with high financial cost, technical difficulties, progressive deterioration of the lung function, and high mortality [18–20]. Shigemura et al. [21] reported that secondary severe pulmonary hypertension, prolonged cardiopulmonary bypass, and high transfusion requirements occurred after LVRS. However, the Canadian Lung Volume Reduction Surgery Group (CLVRS) evaluated patients at 2, 4, 6, and 8 years after LVRS and observed a potential long-term benefit in the survival rates [17, 22]. In addition, NETT studies evaluated patients from 90 days to 4 years of follow-up and concluded that LVRS is an effective treatment option for severe emphysema [2, 23].

Various experimental models of emphysema have been developed, such as fume inhalation, intratracheal instillation of elastase, and genetic modification [24–26]. Although the inhalation of cigarette smoke is the model that most closely resembles human pulmonary emphysema, it requires a long period of exposure to produce similar effects in animals [27]. Another disadvantage is that pulmonary emphysema disappears few weeks after ceasing cigarette smoke exposure [28]. In this study, we opted for the tracheal instillation of a single dose of porcine pancreatic elastase in rats due to the rapid, significant, and permanent enlargement of the air spaces followed by the accumulation of neutrophils and macrophages in the lung [25].

The elastase doses used in this emphysema model widely vary from 2 to 300 UI/100 g of body weight, depending on the subspecies (Brown Norway, Fisher, Lewis, Sprague Dawley and Wistar) [5, 29–36]. Aiming to establish the ideal elastase dose to obtain moderate emphysema, we conducted a pilot study with doses of 3 and 5 UI/100 g. The average Lm at a dose of 5 UI was higher (95 μm) than with 3 UI (75 μm). Furthermore, we evaluated all pulmonary lobe architecture and verified a proportional distribution of elastase action. These findings were similar to those of Emami et al. [32].

Several morphometric methods have been used in experimental models to verify the presence and severity of pulmonary emphysema. Lm analysis is the most commonly used, followed by elastic and collagen fibers quantification [10, 14, 29, 32, 37, 38]. In our study, elastase-treated rats showed Lm values similar to those in other studies [29, 32], indicating the possibility of obtaining a moderate emphysema model with lower doses of elastase. In addition, all animals treated with elastase showed lower Lm values after LBX, indicating an acute effect of this surgery by expanding functional areas of the emphysematous lung.

The elastic fibers arrangement in pulmonary parenchyma is responsible for providing elasticity to the lungs, whereas the collagen fibers are only required in parenchyma distention [39]. In the present study, the emphysematous animals underwent LBX showed increased percentage of elastic fibers, indicating that there was remodeling as a consequence of the LBX. Saldiva et al. [40] demonstrated the increase of collagen fibers in the lung in acute and chronic diseases. In our study, all elastase-treated animals showed a higher proportion of collagen. However, we did not observe any reduction in this proportion up to two weeks after LBX, probably because this period of analysis is insufficient to detect a significant difference.

Several animal models have been used to study CLG. Le Cras et al. [8] observed that CLG occurred only after the resection of three lobes from right lung. Fernandez et al. [9] compared pneumonectomy (PNX) and bilobectomy+pneumonectomy (LBX+PNX) and found no difference in lung weight, but the volume of lower+cardiac lobes was higher in PNX+LBX. Kim et al. [30] showed that emphysematous animals had a 35% greater lung volume than control animals ten months after surgery. In addition, Shigemura et al. [5] resected only the right lower lobe and observed that animals treated with a high dose of elastase had a lower CLG than those treated with saline. They also found that CLG was suppressed in emphysematous lungs after surgical resection, which was associated with a decrease in hepatocyte growth factor.

In the present study, we decided to resect both the right middle and cardiac lobes, which represent 35% of the total lung volume in rats [41]. Animals that underwent bilobectomy (Sal+LBX and Ela+LBX) showed a significant CLG. Based upon other authors (Kaza et al. 2002; Fernandes et al. 2007), we considered that this CLG occurred by hypertrophy and hyperplasia since our results showed higher values of lung weight and volume, alveolar surface density and absolute surface area. Some works have been done to examine the CLG after PNX with the addition of growth factors. Kaza et al. [13] administered keratinocyte growth factor to PNX animals and observed an increase in lung weight and volume, Sv, TVvr, and cellular proliferation in both PNX and PNX+keratinocytes groups. Investigating the retinoic acid effect after PNX, Kaza et al. [42] also observed an increase in several parameters, such as weight and volume of the lungs, alveolar proliferation, TVra, and TVvr. In addition, the transplantation of type II alveolar cells stimulated regeneration during CLG, increasing the weight and volume of the lung [43]. These results reinforce the idea that there is an increase in the respiratory area irrespective of the stimulus.

Growth factors such as VEGF are essential not only for lung parenchymal and vascular development [44, 45], but also for tissue regeneration and vasculature maintenance [26]. Braber et al. [28] compared mice exposed to cigarette smoke for 20 weeks with mice exposed for 20 weeks + 8 weeks without exposure. The results showed a high expression of VEGF in the BALF of both groups. Konerding et al. [46] evaluated the post-LBX CLG in mice and found that the cardiac lobe had higher weight and vascular heterogeneity in the subpleural region. In addition, they found increased expression of angiogenesis-associated factors VEGF alpha and GLUT1. In our study, there was no difference in VEGF levels in lung tissue, but only a trend of higher expression in the Ela+Sham group.

The chronic inflammation of emphysema is characterized by the accumulation of neutrophils, macrophages, and B and T lymphocytes, particularly in small airways [47]. According to the GOLD, inflammation increases proportionally with the disease severity [1]. Inoue et al. [33] reported increased number of total cell and macrophages 21 days after instillation of elastase. Mineo et al. [48] evaluated 28 patients with severe emphysema treated either by LVRS or pulmonary rehabilitation for 12 months and found no difference between groups in relation to inflammatory cells. Our results show a significant decrease in the PMN in lung tissue of the Ela+LBX animals, but only a trend in decreasing total number of cells, macrophages, and neutrophils in the BALF.

Secondary pulmonary hypertension in patients with emphysema may occur as result of both disease and LVRS. Mice exposed to tobacco smoke for 12 or 24 weeks presented hypertension verified by increased right ventricular systolic pressure [38]. Fernandez et al. [9] found that pulmonary hypertension was proportional to the amount of lung mass excised. In addition, Polonio et al. [49] showed that rats which underwent pneumonectomy and treatment with monocrotaline developed hypertension. We used an indirect technique to quantify the index of right ventricular mass relative to body mass and observed an increase in this index only in the Saline+LBX group.

The changes in lung function are a major feature of emphysema. However, the pulmonary function tests in experimental models are less sensitive than morphometry because they only detect severe degrees of airway remodeling or destruction of parenchyma [26]. We have found only few studies in the literature assessing respiratory mechanics data in experimental models of emphysema in rats. Using high doses of elastase (20 and 160 IU), Inoue et al. [33] did not find any difference in lung mechanics in Fischer rats. In contrast, Brewer et al. [29] administered only 2 IU of elastase to Sprague Dawley rats and observed high airway resistance in the emphysematous animals. In our model using Wistar rats and 5 UI of elastase, there was no difference among groups but just a trend of decreased airway resistance in the Ela+LBX group. Further investigation of pulmonary function needs to be performed before reaching a consensus from different emphysema models.

Our results reinforce the importance of the LBX in elastase-treated animals for improving respiratory conditions by increasing the absolute surface area. Moreover, there was a reduction in inflammatory processes in lung tissue. A limitation of this work is the lack of stereological analyses, which could allow for more accurate estimations of various morphological parameters of the lung. In addition, an early VEGF measurement could give more information about the neoalveolization.

## Conclusions

This model of lung resection caused CLG by increasing the absolute surface area. Our morphometry data suggest that there was alveologenesis after LBX in both saline- and elastase-treated animals.

## Supporting information

S1 Fig Experimental groups and study design. (*A*) Saline solution groups (600 μL) were randomized into two subgroups: Sal+Sham and Sal+LBX, with 8 animals per group. Elastase solution groups received 5 UI diluted in 600 μL of saline and were randomized into two groups: Ela+Sham and Ela+LBX, also with 8 animals per group. (*B*) Experimental timeline: (*1*) Six weeks before surgery: saline or elastase solution instillation; (*2*) Six weeks after instillation: sham or LBX surgery with resection of the middle and cardiac lobes; (3) Two weeks after surgery: euthanasia and data collection.
(TIF)

S2 Fig Work-flow scheme of the lung analysis. The right middle and cardiac lobes were excised (LBX). The left lung was used for bronchoalveolar lavage fluid. The right lower lobe was used for lung weight and volume, histological, and immunohistochemistry analysis. The right upper lobe was used for western blot analysis.
(TIF)

S3 Fig VEGF expression and respiratory mechanic. (*A*) VEGF expression, p > 0.05. (*B*) Lung resistance and (*C*) lung elastance, p > 0.05. Data are presented as mean ± standard deviation.
(TIF)

S1 Table Histomorphometry—A 3-level sampling technique adapted from Fernandez et al. [9, 13]. (DOCX)

S2 Table Arterial blood gases analysis. There was no difference in the parameters.
(DOCX)
