# Supplementary figures and images for: Compensatory lung growth after bilobectomy in emphysematous rats
Source: PLoS One. 2017 Jul 27;12(7):e0181819. doi: 10.1371/journal.pone.0181819 (PMC5531597; doi:10.1371/journal.pone.0181819)

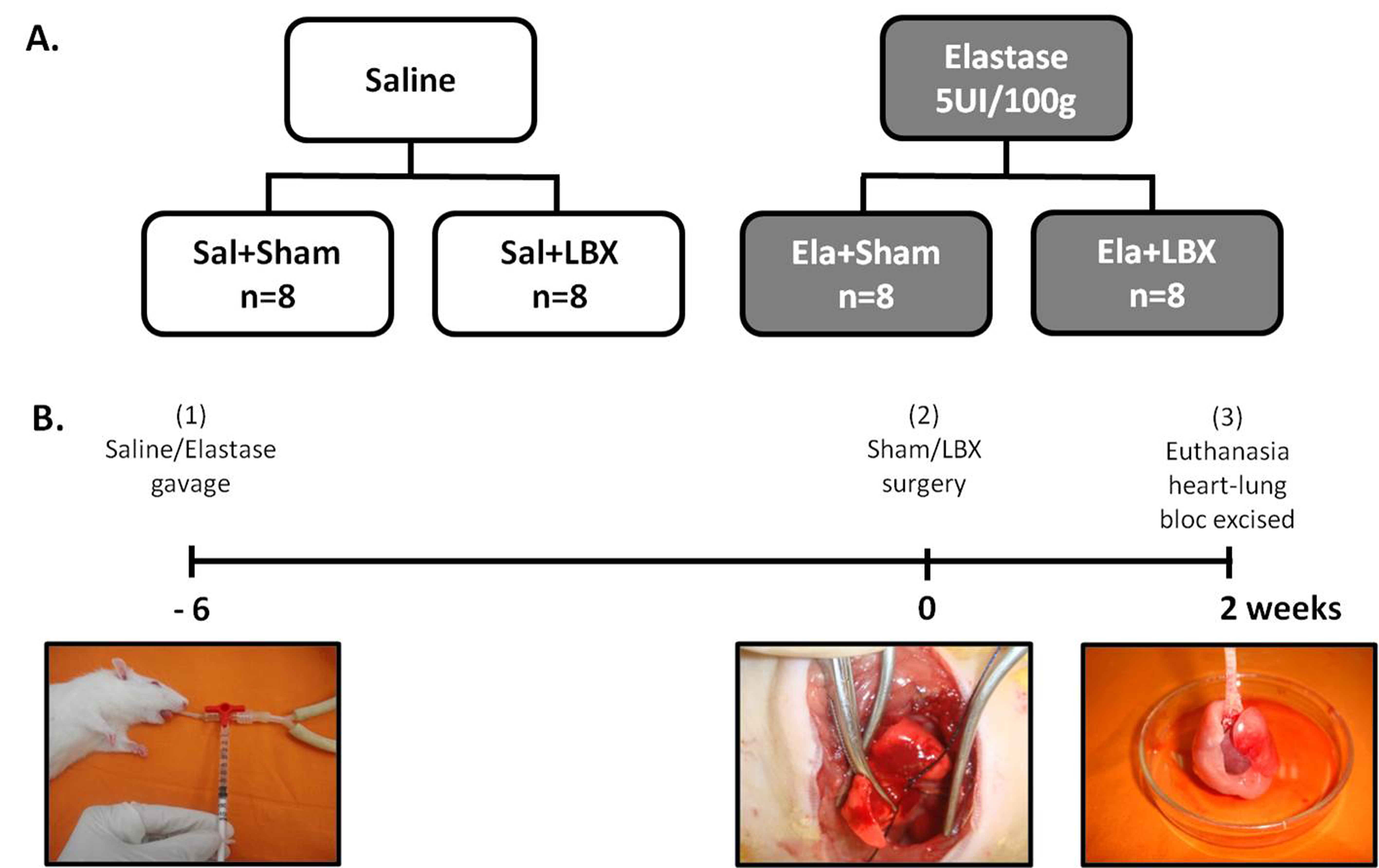

Supplement: S1 Fig — (A) Saline solution groups (600 μL) were randomized into two subgroups: Sal+Sham and Sal+LBX, with 8 animals per group. Elastase solution groups received 5 UI diluted in 600 μL of saline and were randomized into two groups: Ela+Sham and Ela+LBX, also with 8 animals per group. (B) Experimental timeline: (1) Six weeks before surgery: saline or elastase solution instillation; (2) Six weeks after instillation: sham or LBX surgery with resection of the middle and cardiac lobes; (3) Two weeks after surgery: euthanasia and data collection. (TIF) [file pone.0181819.s001.tif]

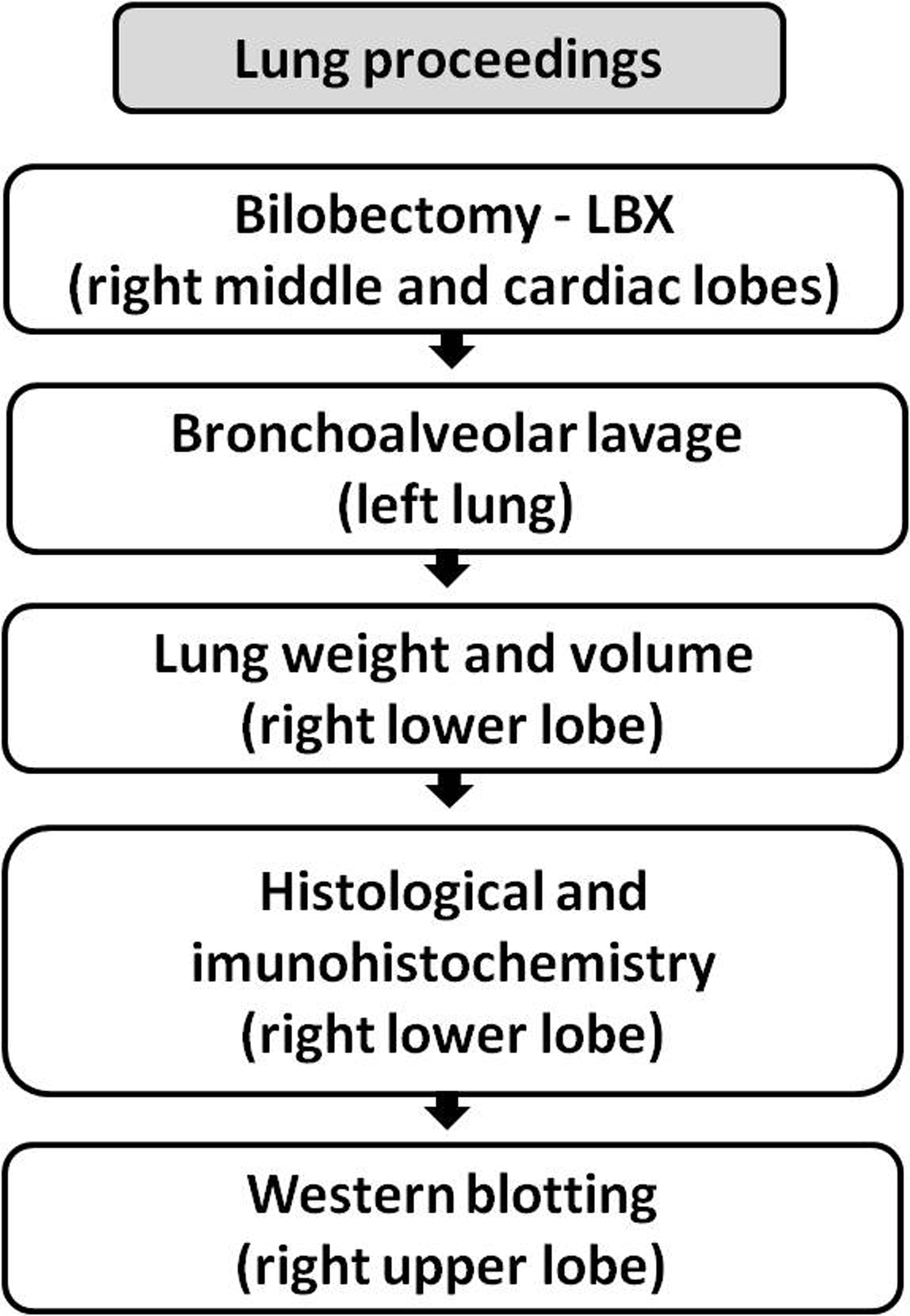

Supplement: S2 Fig — The right middle and cardiac lobes were excised (LBX). The left lung was used for bronchoalveolar lavage fluid. The right lower lobe was used for lung weight and volume, histological, and immunohistochemistry analysis. The right upper lobe was used for western blot analysis. (TIF) [file pone.0181819.s002.tif]

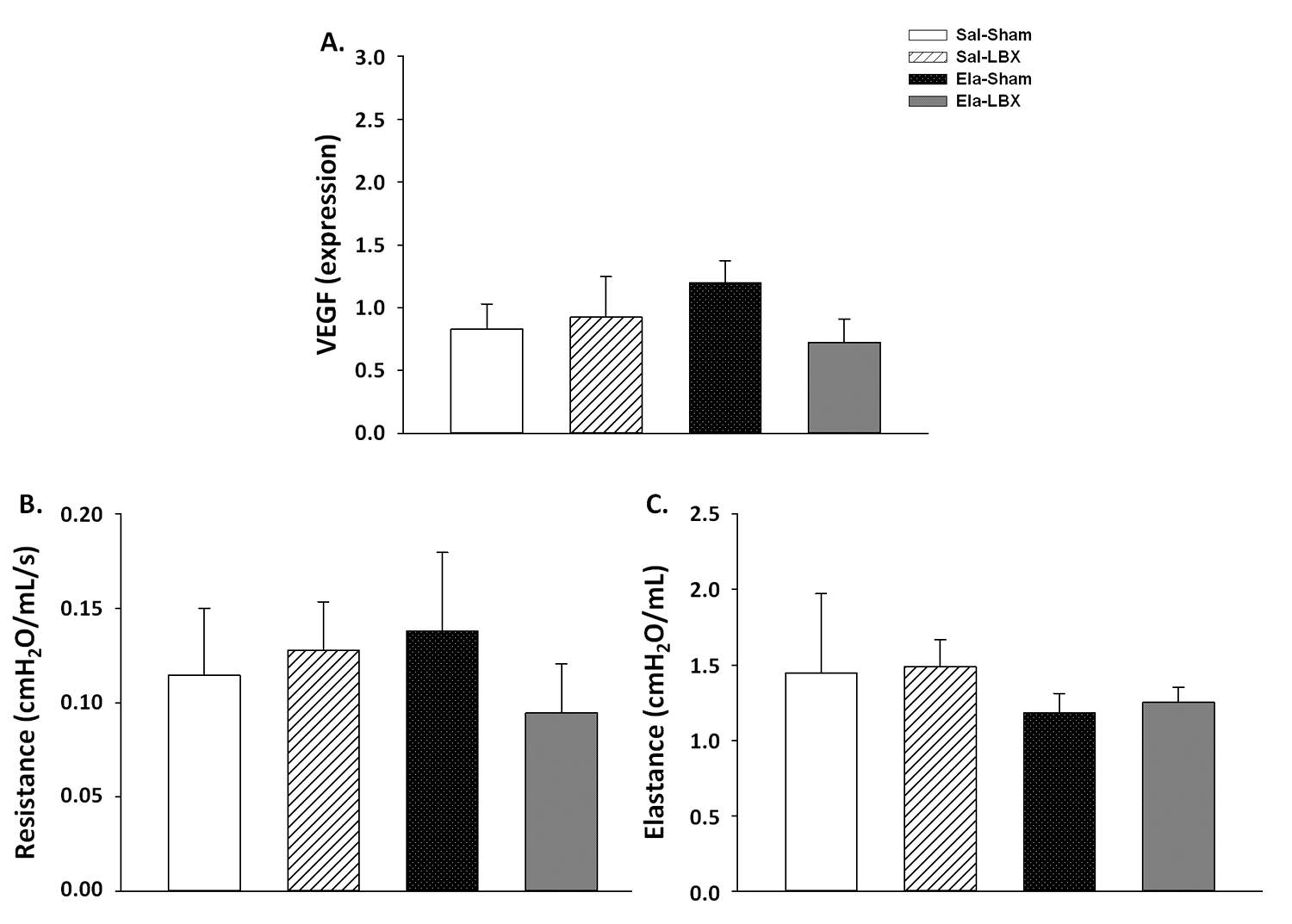

Supplement: S3 Fig — (A) VEGF expression, p > 0.05. (B) Lung resistance and (C) lung elastance, p > 0.05. Data are presented as mean ± standard deviation. (TIF) [file pone.0181819.s003.tif]
