# Supplementary material for: Compensatory lung growth after bilobectomy in emphysematous rats
Source: PLoS One. 2017 Jul 27;12(7):e0181819. doi: 10.1371/journal.pone.0181819 (PMC5531597; doi:10.1371/journal.pone.0181819)
Supplement: S1 Table — (DOCX) [file pone.0181819.s004.docx]

**S2 Table. Histomorphometry - A 3 level sampling technique adapted from Fernandez et al. [9, 13].**

| Level  (magnification) | Parameter | Equation | Specification |
| --- | --- | --- | --- |
| 1  (Gross inspection) | Obs.: Do not performed since the small size of the rat lung does not permit an accurate differentiation at the gross level. | | |
| 2  (50x) | Volume density of respiratory region | Vvr=Pr/100 | Pr = number of lattice points on intra-acinar air space |
| 3  (200x) | Volume density of respiratory airspace | Vra=Pra/100 | Pra = number of lattice points on respiratory airspace |
|  | Volume density of respiratory tissue | Vrt=Prt/100 | Prt = number of lattice points on respiratory tissue |
|  | Density of alveolar surface | Sv=2/d x (Is/Pp) | d = length of the lattice grid line  Is = number of lattice test lines intercepted by airspace– epithelial interfaces  Pp = number of lattice points on lung parenchyma |
|  | Total volume of respiratory region | TVvr=Vvr x VI | Vvr = volume density of respiratory region  Vl = total lung volume |
|  | Total volume of respiratory airspace | TVra= Vra x VI | Vra = volume density of respiratory airspace  Vl = total lung volume |
|  | Total volume of respiratory tissue | TVrt= Vrt x VI | Vrt = volume density of respiratory tissue  Vl = total lung volume |
|  | Absolute surface area | Sv.TVvr | Sv= density of alveolar surface  TVvr= total volume of respiratory region |
