# Supplementary material for: Compensatory lung growth after bilobectomy in emphysematous rats
Source: PLoS One. 2017 Jul 27;12(7):e0181819. doi: 10.1371/journal.pone.0181819 (PMC5531597; doi:10.1371/journal.pone.0181819)
Supplement: S2 Table — There was no difference in the parameters. (DOCX) [file pone.0181819.s005.docx]

**S4 Table. Arterial blood gases analysis.**

|  | pH  Mean±DP | *p*CO_2_  (mmHg)  Mean±DP | *p*O_2_  (mmHg)  Mean±DP | Hct  (%)  Mean±DP | *s*O_2_  (%)  Mean±DP | Lac  (mmol/mL)  Mean±DP |
| --- | --- | --- | --- | --- | --- | --- |
| Sal+Sham | 7.5 ± 0.1 | 19.1 ± 3.7 | 96.1 ± 19.4 | 40.3 ± 2.8 | 98.1 ± 0.5 | 4.6 ± 1.3 |
| Sal+LBX | 7.5 ± 0.0 | 18.6 ± 1.9 | 101.2 ± 23.5 | 39.9 ± 3.7 | 98.4 ± 1.1 | 4.6 ± 1.1 |
| Ela+Sham | 7.5 ± 0.0 | 20.6 ± 3.1 | 105.7 ± 22.3 | 40.0 ± 1.7 | 98.0 ± 1.7 | 3.8 ± 1.0 |
| Ela+LBX | 7.5 ± 0.1 | 19.6 ± 1.6 | 128.9 ± 25.1 | 40.7 ± 1.7 | 99.3 ± 0.4 | 4.8 ± 1.0 |

There was no difference in the parameters.
